# β-Catenin Loss in Hepatocytes Promotes Hepatocellular Cancer after Diethylnitrosamine and Phenobarbital Administration to Mice

**DOI:** 10.1371/journal.pone.0039771

**Published:** 2012-06-25

**Authors:** Prince Kwaku Awuah, Byung Han Rhieu, Sucha Singh, Amalea Misse, Satdarshan P. S. Monga

**Affiliations:** 1 Department of Pathology, University of Pittsburgh, Pittsburgh, Pennsylvania, United States of America; 2 Department of Medicine, University of Pittsburgh, Pittsburgh, Pennsylvania, United States of America; University of North Carolina School of Medicine, United States of America

## Abstract

Hepatocellular Carcinoma (HCC) is the fifth most common cancer worldwide. β-Catenin, the central orchestrator of the canonical Wnt pathway and a known oncogene is paramount in HCC pathogenesis. Administration of phenobarbital (PB) containing water (0.05% w/v) as tumor promoter following initial injected intraperitoneal (IP) diethylnitrosamine (DEN) injection (5 µg/gm body weight) as a tumor inducer is commonly used model to study HCC in mice. Herein, nine fifteen-day male β-catenin knockout mice (KO) and fifteen wild-type littermate controls (WT) underwent DEN/PB treatment and were examined for hepatic tumorigenesis at eight months. Paradoxically, a significantly higher tumor burden was observed in KO (p<0.05). Tumors in KO were β-catenin and glutamine synthetase negative and HGF/Met, EGFR & IGFR signaling was unremarkable. A significant increase in PDGFRα and its ligand PDGF-CC leading to increased phosphotyrosine-720-PDGFRα was observed in tumor-bearing KO mice (p<0.05). Simultaneously, these livers displayed increased cell death, stellate cell activation, hepatic fibrosis and cell proliferation. Further, PDGF-CC significantly induced hepatoma cell proliferation especially following β-catenin suppression. Our studies also demonstrate that the utilized DEN/PB protocol in the WT C57BL/6 mice did not select for β-catenin gene mutations during hepatocarcinogenesis. Thus, DEN/PB enhanced HCC in mice lacking β-catenin in the liver may be due to their ineptness at regulating cell survival, leading to enhanced fibrosis and regeneration through PDGFRα activation. β-Catenin downregulation also made hepatoma cells more sensitive to receptor tyrosine kinases and thus may be exploited for therapeutics.

## Introduction

Hepatocellular Carcinoma (HCC) is the fifth most common cancer and the third cause of cancer death worldwide [1]. There is a strong need to delineate the molecular alterations responsible for the initiation and exacerbation of this disease. In order to study the cellular and molecule perturbations in HCC, many preclinical strategies employ the use of genetic and chemical models of carcinogenesis. Administration of diethylnitrosamine (DEN) alone or in conjunction with phenobarbital (PB) in mice is frequently used to induce HCC in mice.

One pathway of critical importance in HCC is the Wnt/β-catenin signaling. β-Catenin is the central effector of the canonical Wnt signaling, which is a highly conserved pathway regulating critical cellular processes such as proliferation, differentiation, survival and self-renewal [2], [3], [4], [5]. In the absence of Wnt, β-catenin is phosphorylated at amino-terminal serine and threonine residues and targeted for ubiquitination [6]. Upon binding of Wnt protein to its cell surface receptor Frizzled and co-receptor low-density lipoprotein– related protein 5/6 (LRP5/6), a signal is transduced through disheveled that allows for inactivation of degradation complex comprised of glycogen synthase kinase 3β (GSK3β), adenomatous polyposis coli gene product (APC) and casein kinase Iα, which allows β-catenin to dissociate and translocate to the nucleus to bind to lymphoid enhancer-binding factor/T cell factor (LEF/TCF) family of proteins to transactivate target genes. The Wnt/β-catenin pathway has been implicated in a subset of HCC where activating mutations in the β-catenin gene (*CTNNB1*) have been reported in 20%–40% of patients [7], [8]. Knockdown of β-catenin in hepatoma cells leads to decreased growth and survival. For the aforementioned reasons, β-catenin is a well-recognized oncogene and considered a valuable therapeutic target.

With this background, we hypothesized that lack of β-catenin in hepatocytes might protect against chemical-induced carcinogenesis especially in a model where HCC is conceived through tumor induction by DEN and tumor promotion through the continuous use of PB [9], [10]. We used male conditional hepatocyte-specific β-catenin knockout (KO) mice and sex-matched wild-type littermate controls (WT) to study tumorigenesis in response to DEN/PB. We report a paradoxical increase in hepatic tumorigenesis in the absence of β-catenin that was attributable to enhanced injury, fibrosis and ensuing regeneration, which appear to be driven by a rather non-classical epithelial receptor tyrosine kinase receptor PDGFRα. We also demonstrate that utilizing the commonly employed DEN/PB protocol in C57BL/6 mice, the tumorigenesis does not occur through β-catenin mutations as is observed in C3H mice. Lastly, β-catenin inhibition lead to PDGFRα activation and thus may make the hepatoma cells more amenable to receptor tyrosine kinases inhibition for therapies.

## Results

### β-Catenin loss in hepatocytes engenders enhanced hepatocarcinogenesis in mice in response to DEN/PB

**Figure 1:**
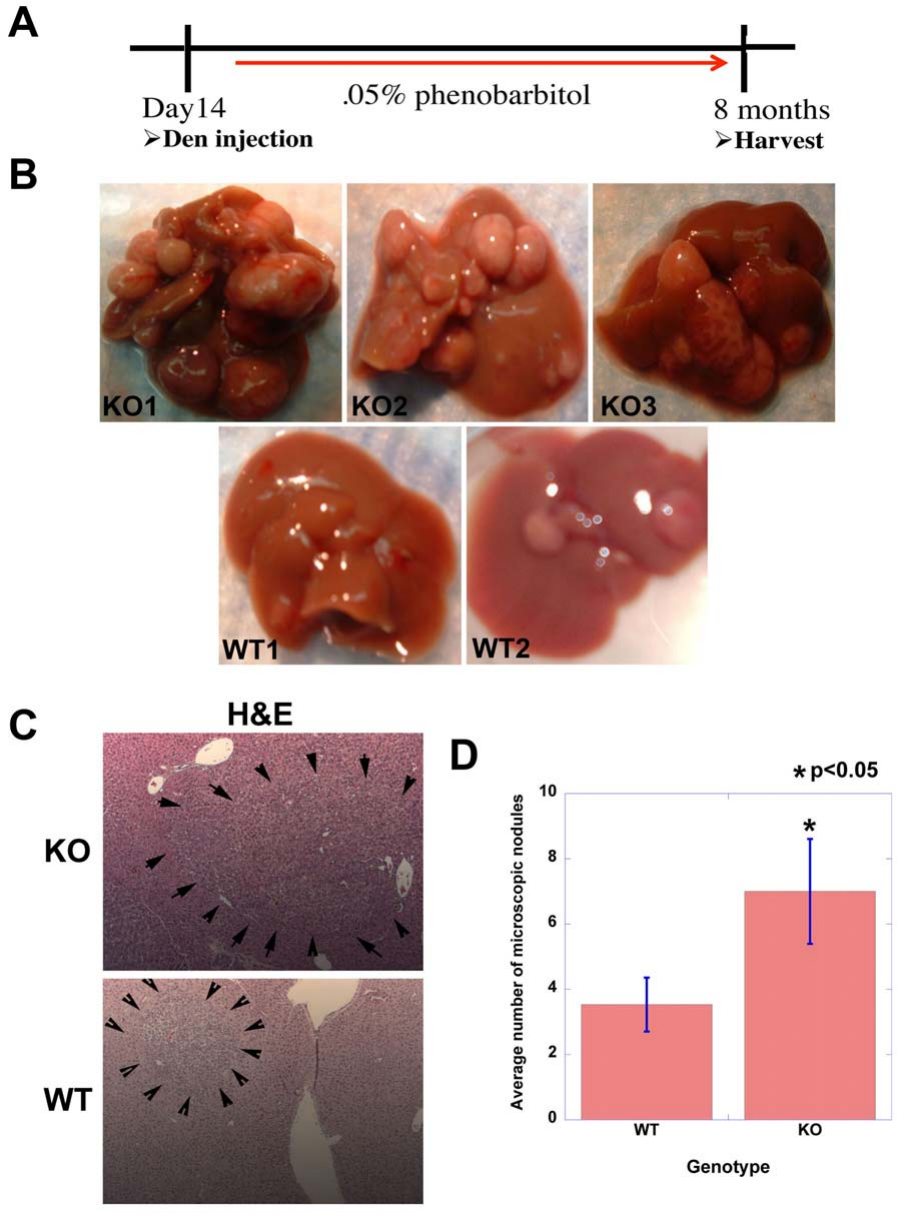
Enhanced tumorigenesis in β-catenin KO mice exposed to DEN/PB regimen. Experimental strategy summarizing DEN/PB treatment in KO and WT mice. A. Representative photographs of tumor-bearing livers in DEN/PB treated KO and WT mice at the time of harvest at 8 months of age. B. DEN/PB induced microscopic tumor foci (outlined by arrowheads) visualized by H&E in WT and KO livers at 8 months of age. C. A significant increase in microscopic tumor foci in KO as compared to WT (p<0.05). Tumors were counted from H&E stained sections representing 4 liver lobes from each KO and WT animals on DEN/PB protocol.

WT and KO male mice (C57BL/6) were given a single dose (5 µg/gram) of DEN injection at postnatal day 14 days and two weeks later allowed *ad libitum* access to PB containing drinking water for 8 months at which time mice were examined for liver tumors (Fig. 1A). Intriguingly, the mice lacking β-catenin in hepatocytes displayed significantly enhanced tumorigenesis than WT mice that was grossly appreciable as larger and greater numbers of tumors (Fig. 1B). H&E staining was employed to also determine the microscopic tumor foci in both groups of animals (Fig. 1C). The total numbers of foci were counted in representative sections from four lobes from the KO and WT, which show significantly more tumors in KO as compared to the WT (p<0.05) (Fig. 1D).

### β-Catenin KO livers after DEN/PB treatment shows increased cell death, stellate cell activation and fibrosis and tumor proliferation

**Figure 2:**
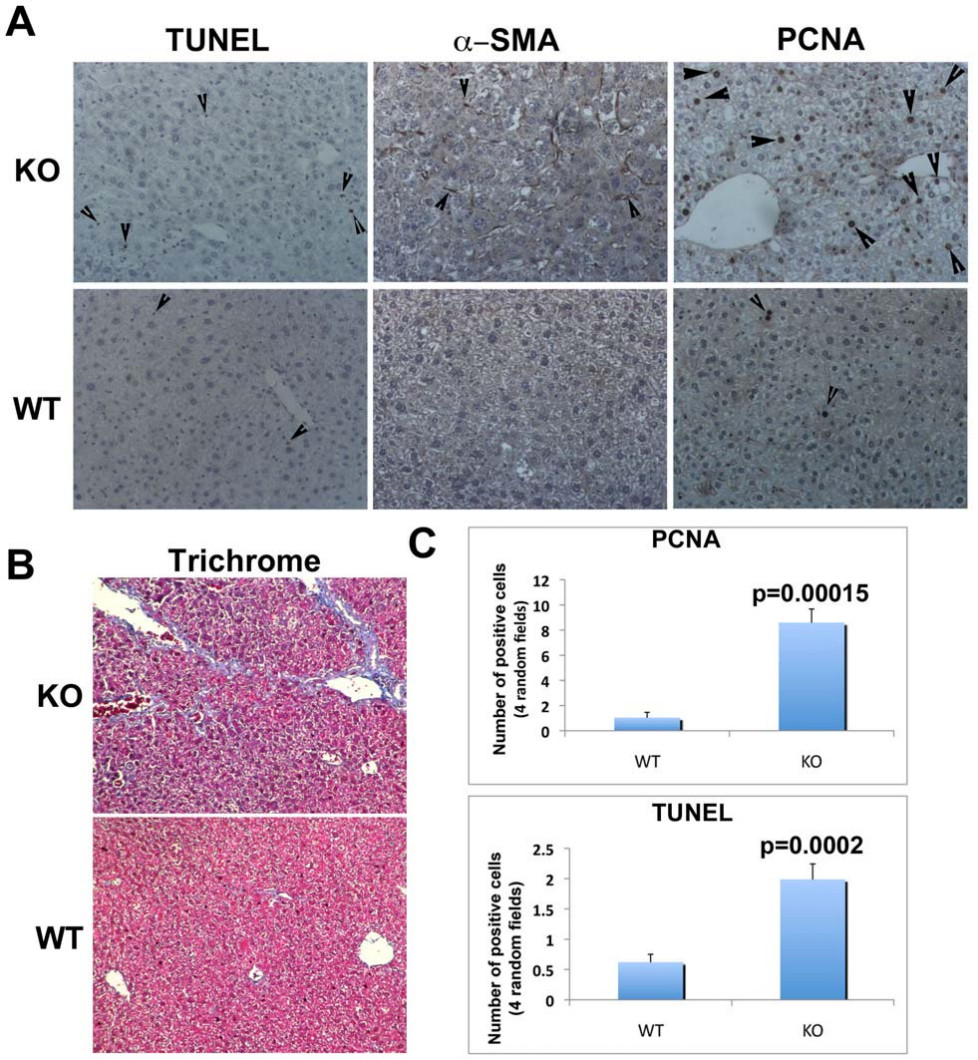
Tumorigenesis in KO mice following DEN/PB treatment is associated with greater injury, fibrosis and regeneration. A. Representative tumor-bearing KO livers show increased parenchymal cell death (TUNEL), greater stellate cell activation (α-SMA) and increased parenchymal cell proliferation (PCNA) as compared to WT. B. Increased hepatic fibrosis (blue) in tumor-bearing KO livers is evident by Masson Trichrome staining as compared to control. C. PCNA- and TUNEL-positive cells were counted in 4 random sections of 5 representative KO and WT livers. A significant increase in both PCNA and TUNEL positive parenchymal cells was evident in KO livers as compared to WT after DEN/PB treatment.

Next, we addressed the cellular mechanisms that may be the basis of enhanced tumorigenesis in KO. We identify higher numbers of TUNEL-positive hepatocytes in KO at 8 months after DEN/PB as compared to similarly treated WT, suggesting greater cell death (Fig. 2A). There was an accompanying increase in hepatic parenchymal cell proliferation in KO that exceeded that of WT (Fig. 2A). In addition, KO mice exhibited a dramatic increase in the numbers of α-smooth muscle actin, which identifies activated stellate cells that are responsible for collagen deposition and fibrosis (Fig. 2A). Concomitant to stellate cell activation, we observed enhanced fibrosis by Masson Trichrome staining in the KO as compared to the WT (Fig. 2B). In fact only one of the 15 WT animals after DEN/PB challenge showed fibrosis, which was comparable to fibrosis in the KO. All together, our results suggest that greater tumorigenesis in KO livers was associated with significantly greater cell death and proliferation (Fig. 2C), and hepatic fibrosis as well.

### Tumors in KO livers after DEN/PB are not composed of β-catenin-positive hepatocytes

**Figure 3:**
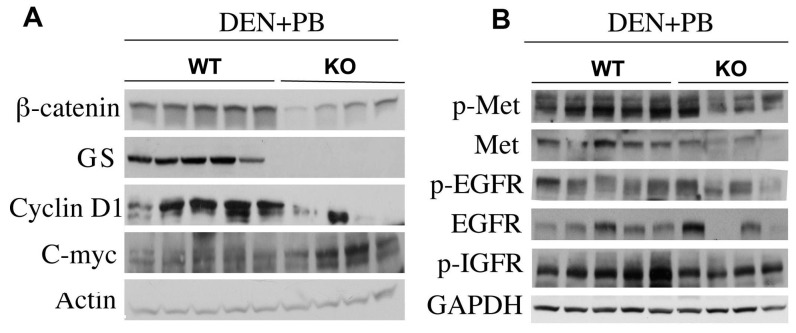
Molecular signaling in tumor-bearing KO and WT mice. A. Representative western blot analysis from 5 WT and 4 KO tumor-bearing livers shows low β-catenin, absent GS and dramatically lower cyclin-D1 in KO whereas c-Myc levels were increased. Actin verifies equal loading. B. Examination of RTK in the same sets of animals shows notably lower phospho-Met and phospho-IGFR and only marginally lower phospho-EGFR, in KO than WT livers by western blots. GAPDH verifies comparable loading.

**Figure 4:**
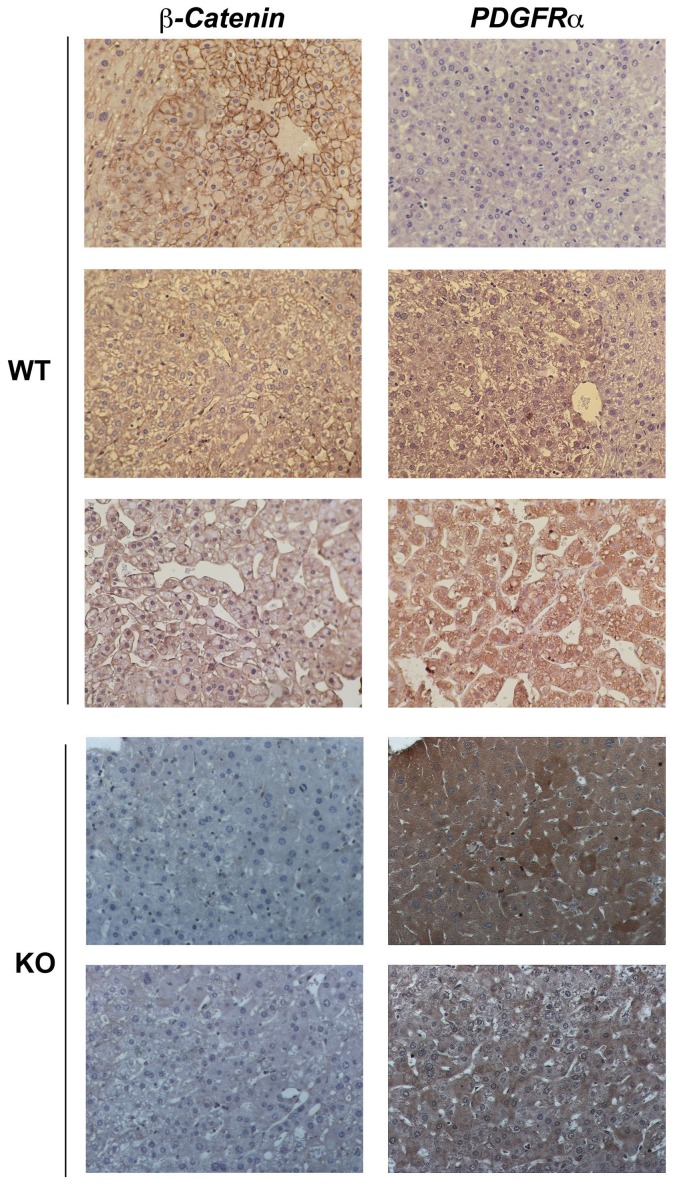
β-Catenin and PDGFRα immunohistochemistry in tumors in WT and KO exposed to DEN/PB. Tumors in WT were heterogeneous and were either positive for both β-catenin and PDGFRα, or for either one of them. In KO, almost all tumors lacked any β-catenin and showed intense PDGFRα-positivity.

Since there have been reports from our lab and others that in response to specific injuries, there is pressure on hepatocytes that may have escaped cre-mediated deletion, our first goal was to ascertain if livers exposed to DEN/PB from KO group showed any reappearance of β-catenin especially when compared to the WT. A representative WB showed that all KO livers from DEN/PB exposed mice expressed dramatically lower levels of β-catenin as compared to WT by western blots (Fig. 3A). This was also verified by a detailed immunohistochemical analysis included in a forthcoming section (Table 1 and Fig. 4). The low level of β-catenin in KO livers represents its presence in the non-parenchymal cells of the liver that show no albumin cre-mediated recombination. Similarly analysis presented from representative KO and WT livers also showed absence of glutamine synthetase, a surrogate target gene of β-catenin signaling in the KO (Fig. 3A). Cyclin-D1, another prominent target of β-catenin in liver and elsewhere was increased in most WT, whereas 8/9 KO showed very low or absent cyclin-D1 in response to DEN/PB (Fig. 3A and not shown). C-Myc, another target of β-catenin, was ironically higher in KO as compared to WT mice after DEN/PB exposure as shown in a representative western blot (Fig. 3A). Thus KO livers after DEN/PB continue to be negative for β-catenin after 8 months.

**Table 1:** Summary of immunohistochemical findings of microscopic tumor foci for β-catenin and PDGFRα.

SAMPLE	NUMBER OF TUMOR FOCI	β-CATENIN-POSITIVE FOCI		PDGFRα-POSITIVE FOCI	NUMBER OF FOCI POSITIVE FOR BOTH N/C	FIGURE 4
		N/C	M	C	β-CATENIN & PDGFRα	
WT1	2	0	1	0	0	
WT2	2	0	0	2	0	
WT3	0	0	0	0	0	
WT4	4	4	3	2	2	
WT5	0	0	0	0	0	
WT6	9	2	1	6	1	
WT7	2	0	1	0	0	
WT8	6	5	5	3	3	
WT9	4	1	4	1	0	X
WT10	2	2	2	2	2	X
WT11	6	0	6	4	0	
WT12	2	0	2	1	0	
WT13	2	1	2	2	1	
WT14	11	2	8	7	1	X
WT15	1	0	1	0	0	
	***55***	***17***	***36***	***30***	***9***	
KO1	17	1	0	17	1	X
KO2	11	0	0	10	0	
KO3	3	0	0	3	0	X
KO4	4	0	0	4	0	
KO5	2	0	0	2	0	
KO6	6	0	0	6	0	
KO7	6	1	0	4	1	
KO8	10	0	0	10	0	
KO9	4	0	0	3	0	
	***63***	***2***	***0***	***59***	***2***	

Abbreviations: WT-wildtype; KO-knockout; N/C- nuclear/cytoplasmic; M-membranous; C-cytoplasmic.

*Tumors in KO livers after DEN/PB are not associated with activation of traditional HCC associated receptor tyrosine kinases.* Due to a paradoxical increase in DEN/PB-induced tumorigenesis in KO, we next explored possible molecular mechanisms. Both epidermal growth factor (EGF) signaling and hepatocyte growth factor (HGF) signaling are critical players in the development and exacerbation of HCC [11]. We explored both total and phosphorylated status of receptors of these receptor tyrosine kinases (RTKs) in livers from DEN/PB-treated KO and WT mice. Interestingly we noted a decrease in total and phosphorylated levels of c-Met, the HGF receptor in KO animals as compared to WT (Fig. 3B). Both total and phosphorylated levels of EGFR between WT and KO animals remain unaltered (Fig. 3B). We also examined insulin like growth factor receptor, another signaling pathway implicated in HCC [11]. We noted higher levels of its phosphorylation in the WT as compared to KO (Fig. 3B). Thus, classical RTKs do not appear to be responsible for enhanced HCC in KO but p-Met and P-IGFR are prominently induced in tumor bearing WT mice indicating their important role in HCC.

### Immunohistochemical characterization of KO and WT liver tumors for β-catenin and PDGFRα

We next characterized the tumors observed in the WT and KO by immunohistochemistry for β-catenin localization. Around 31% of total tumor foci in WT mice showed nuclear β-catenin (Table 1, Fig. 4). Out of 63 observed foci in 9 KO mice, only two were composed of β-catenin-positive tumor cells that exhibited its nuclear/cytoplasmic localization (data not shown) while others were negative (Fig. 4). This substantiated the observations in Fig. 3A and also demonstrates that almost all tumors in this group were comprised by β-catenin-negative hepatocytes and cannot be due to expansion of cells, which may have retained β-catenin due to incomplete albumin-cre recombination.

We next investigated PDGFRα signaling, which has been implicated in HCC and is involved in tumor growth, angiogenesis, and maintenance of tumor microenvironment [12], [13], [14]. 54.5% of all tumor foci in the WT mice showed cytoplasmic PDGFRα expression (Table 1). We found around 94% of tumor foci in KO to be strongly positive for PDGFRα in the cytoplasm of tumor cells (Table 1, Fig. 4). Only four foci were negative for PDGFRα. The two tumor foci that were β-catenin-positive in the KO livers were simultaneously positive for PDGFRα.

In an additional analysis, 9 of the 55 tumors observed in the WT were concomitantly positive for both PDGFRα and nuclear β-catenin, while others were positive for either one of the two (Table 1, Fig. 4). A small fraction of tumors were negative for both these proteins. The two tumors that were composed of β-catenin-positive hepatocytes in the KO were also positive for PDGFRα signaling (Table 1). Overall, PDGFRα expression was higher and also in greater numbers of tumor foci in the KO as compared to the WT 8 months after DEN/PB exposure.

**Figure 5:**
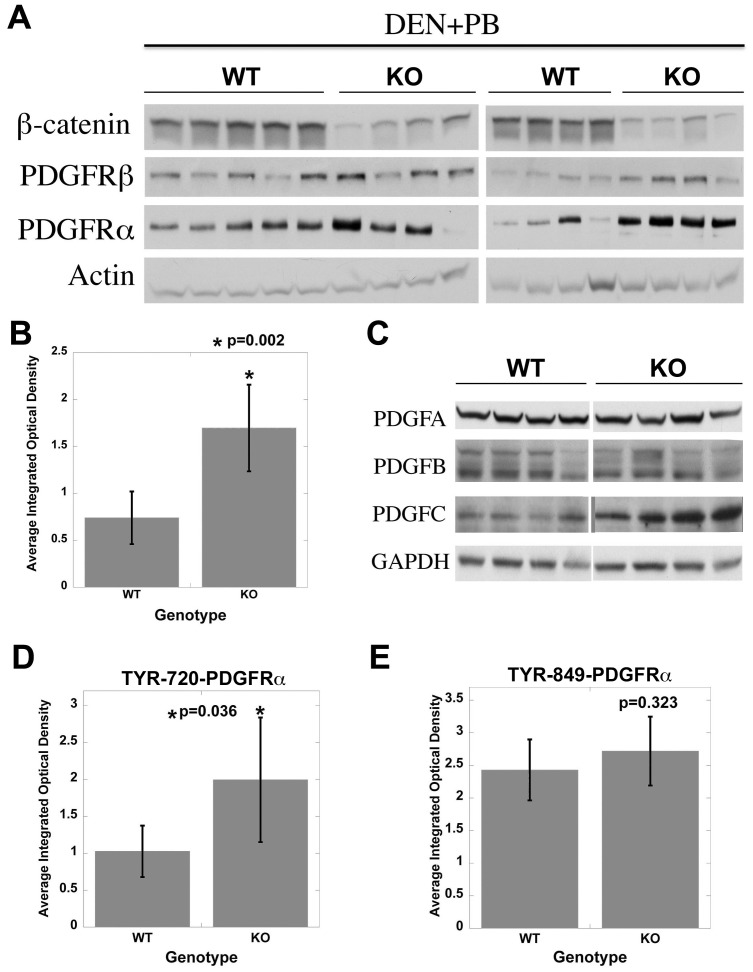
Tumor-bearing KO mice display active PDGFRα signaling when compared to WT. A. Representative western blots from 8 KO and 9 WT show a dramatic increase in total levels of PDGFRα and modest increase in PDGFRβ in the KO. Actin loading verifies equal loading. B. Average integrated optical density (IOD) obtained from scanned autoradiographs shown in Fig. 5A revealed significantly higher PDGFRα levels in KO (p = 0.002). C. Western blot from representative samples shows a dramatic increase in PDGF-CC, a ligand for PDGFRα in KO whereas PDGF-AA and BB remained unremarkable between the two groups. D. Bar graph depicts a significant increase in Tyr720-PDGFRα in KO as compared to WT (p<0.05). E. Insignificant differences were evident in Tyr-849-PDGFRα between the WT and KO.

*DEN/PB induced increased tumorigenesis is associated with activation of PDGFRα signaling.* To further verify PDGFRα increase in the KO over WT after DEN/PB, we utilized western blots analysis. PDGFRα protein levels were higher in the KO than the WT livers (Fig. 5A), and this difference was statistically significant (Fig. 5B). There was a modest increase in PDGFRβ levels in the KO livers (Fig. 5A). We also identified a noteworthy increase in total protein levels of selective PDGFRα ligand PDGF-CC while PDGF-AA or PDGF-BB remained unaltered between the two groups (Fig. 5C).

To determine consequences of enhanced PDGF-CC/PDGFRα levels in KO we assessed levels of PDGFRα phosphorylation at several specific tyrosine residues using antibodies listed in methods. As shown in densitometric analyses, a significant increase in Tyr720-PDGFRα (p<0.05) (Fig. 5D) but not in Tyr849-PDGFRα (Fig. 5E) or Tyr572/574- and Tyr754-PDGFRα (not shown) was observed in the KO. These observations demonstrate PDGFRα activation in KO, which may be playing an important role in hepatocarcinogenesis.

### PDGFRα ligand stimulates hepatoma cell proliferation only upon β-catenin suppression

**Figure 6:**
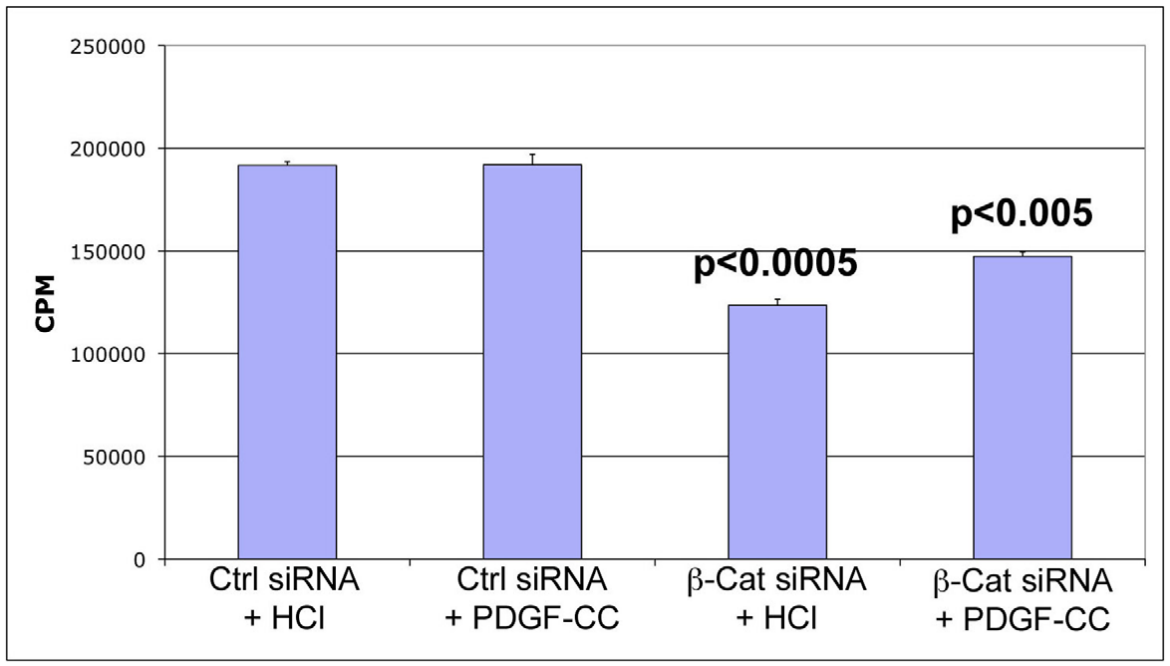
β-Catenin suppression engenders mitogenicity to PDGF-CC in hepatoma cell culture. PDGF-CC (10 ng/ml) treatment does not increase DNA synthesis as compared to HCl treatment of Hep3B cells. β-Catenin knockdown led to significant decrease in thymidine incorporation as compared to control siRNA (p<0.0005). However PDGF-CC treatment led to a significant increase in thymidine incorporation in β-catenin-suppressed as compared to control siRNA-transfected cells (p<0.005).

To further ascertain the relevance of PDGFRα signaling in absence of β-catenin, we utilized human hepatoma cells. PDGF-CC was unable to induce any significant increase in Hep3B cell DNA synthesis when compared to HCl, which was utilized to reconstitute PDGF in almost confluent cell cultures (Fig. 6). β-Catenin knockdown when compared to control siRNA transfection, significantly lowered thymidine incorporation in Hep3B cells (p<0.0005) (Fig. 6). Only upon β-catenin silencing, was PDGF-CC treatment able to induce significant DNA synthesis in Hep3B cells as compared to HCl (p<0.005). Thus β-catenin suppression enabled PDGF-CC to be mitogenic to Hep3B cells.

## Discussion

To understand the molecular and cellular basis of HCC in patients, several preclinical models are in use including DEN or DEN/PB regimens in rodents. DEN is a commonly used carcinogen to induce HCC in rodent models, however it has high strain specificity. In C57BL/129Sv×C3H/He mice, a strain more susceptible to hepatocarcinogenesis, DEN injection at around 6 weeks of age at a dose of 90 µg/gm body weight, induces HCC through Ha-Ras mutations, while inclusion of PB in drinking water after 3 weeks of DEN, promotes tumorigenesis due to *CTNNB1* mutations. [15]. However, another study in male B6C3F1 mice, obtained by interbreeding female C57BL/6J and male C3H/HeJ mice, injected DEN at 10 µg/gm body weight at 3 weeks of age without PB, showed HCC via *CTNNB1* mutations [16]. Another model utilizes DEN at a dose of 5 µg/gm body in C57BL/6 mice, a strain relatively resistant to HCC. Here, DEN induces DNA adducts in hepatocytes undergoing cell division, and eventually leads to development of HCC [17], [18]. Inclusion of PB enhances tumorigenesis via its tumor promoting ability [9], [10]. In our study, we show that unlike C57BL/129Sv×C3H/He mice, DEN/PB induced liver tumors in the C57BL/6 mice did not exhibit nuclear/cytoplasmic localization of β-catenin and hence does not selectively cause HCC via *CTNNB1* mutations. Thus mouse strain consideration for studying tumorigenesis in response to specific protocols is highly relevant.

Many pathways broadly categorized into Ras/MAPK, PIK3CA/AKT, and Wnt/β-catenin signaling, have been shown to be of significance in HCC [11], [19]. β-Catenin, the central orchestrator of Wnt signaling, is a known oncogene due to its implications in a variety of cancers, including 20%–40% of all HCCs [20]. Intriguingly though, overexpression of either wild-type or mutant form of β-catenin in murine livers is unable to induce spontaneous HCC [21], [22], [23], [24]. However, in the presence of another ‘hit’ in the form of a transgene or a chemical carcinogen, these various mice show enhanced tumorigenesis [23], [25], [26]. Paradoxically, conditional loss of β-catenin in hepatocytes in C57BL/6 mice led to an unexpectedly higher susceptibility to DEN-induced HCC [27]. Another group also recently showed demonstrated increased tumorigenesis in KO mice in response to DEN/PB, *albeit* in C3H/N mice [28]. In the current study, we provide evidence that β-catenin KO mice in C57BL/6 background subjected to DEN-mediated tumor induction at P14 followed by tumor promotion 2 weeks later by PB also led to a dramatically higher tumor burden.

DEN or DEN/PB induced HCC in any strain of mice is not typically associated with any hepatic fibrosis. However, in the β-catenin conditional null mice DEN/PB exposure led to development of HCC, which was associated with hepatic fibrosis and is in line with recent studies by others and us [27], [28]. We also saw an increase in cell death and resulting increase in cell proliferation. In fact we identified increased stellate cell activation, a modest increase in PDGFRβ and ensuing hepatic fibrosis. These data indicate that β-catenin loss makes livers more prone to genotoxic injury and eventually tumorigenesis mimicking the predominant scenario of human HCC where tumors often occurs in cirrhotic background [29]. Role of β-catenin in regulating redox state has been implied in many recent studies where its interactions with HIF1α, FOXO3 and others may be critical [30], [31]. It will thus be important to understand the basis of such ‘tumor suppressive’ roles of β-catenin in HCC that may eventually require careful selection of patients to be treated with anti-β-catenin therapies [32].

To ascertain the molecular basis of HCC in the absence of β-catenin, we were interested in signaling pathways that are well known in the development and exacerbation of HCC. C-Met and IGFR phosphorylation was increased in WT but not in KO mice while EGFR activation was only mildly elevated in WT. Thus while these RTK's may be playing an important role in tumorigenesis in the WT exposed to DEN/PB, their participation in KO is unlikely. Based on our previous findings in DEN-induced tumorigenesis in KO [27] and independent studies showing an important role of PDGFR signaling in HCC [12], [13], [14], we investigated its expression and activation in DEN/PB studies. PDGFRα levels were significantly upregulated in KO after DEN/PB-exposure. In addition, PDGF-CC a selective ligand of PDGFRα, whose overexpression in liver-specific transgenic mice has been shown to induce cirrhosis and HCC [33], was also increased in KO. In fact PDGF-CC was localized to hepatocytes in KO exposed to DEN/PB (data not shown) suggesting an autocrine loop of signaling. The results obtained from thymidine incorporation assay in the Hep3B cells, corroborates *in vivo* findings. While decreased cell proliferation was observed in hepatoma cells after β-catenin knockdown [34], PDGF-CC promoted their mitogenesis more robustly only after β-catenin suppression that leads to PDGFRα upregulation [27]. This also verifies PDGFRα signaling as a means of escape from β-catenin therapeutic inhibition.

PDGFRα overexpression in the presence of increased PDGF-CC led to increased Tyr720-PDGFRα in tumor bearing KO but not WT livers after DEN/PB. Phosphorylation at tyrosine 720 is known to activate phosphatase SHP2, which in turn dephosphorylates Src, leading to its activation [35]. Src activation has been shown to induce c-Myc, an important proto-oncogene [36], which was concomitantly elevated in KO. Other tyrosine sites in PDGFRα showed inconspicuous changes in phosphorylation between the KO and WT and thus may be of lesser relevance in the current tumorigenesis model.

Only around 3% of all tumors in the 9 KO's at 8 months after the DEN/PB regimen were β-catenin-positive tumors that may be due to ‘leaky’ cre-recombinase. β-Catenin-negative tumors were also negative for GS and mostly negative for cyclin-D1. We have not followed any animals in this study beyond 8 months. Others have recently reported extensive spontaneous repopulation in KO livers *albeit* at 18–20 months of age [37]. One group has reported a more robust spontaneous repopulation and hepatic adenomatosis in KO, which has not been reported by any other group working with the β-catenin conditional knockout mice [38]. The only instance of extensive repopulation in the KO mice in our experience was observed after continuous administration of diet containing 0.1% 3,5-diethoxycarbonyl-1,4-dihydrocollidine (DDC) for 5 months [39]. In fact KO mice have been studied after partial hepatectomy, methionine-choline deficient diet, alcohol diet or DEN and have exhibited lack of hepatic repopulation with β-catenin-positive hepatocytes [27], [31], [40], [41].

## Materials and Methods

### Animal studies

All animal experiments were performed under the guidelines of the National Institutes of Health and the Institutional Animal Use and Care Committee at the University of Pittsburgh. The studies performed in the current report were approved by the Institutional Animal Use and Care Committee at the University of Pittsburgh. Mice with conditional deletion of β-catenin in hepatocytes with genotype- Ctnnb1^loxp/loxp^;Alb-Cre^+/−^ are refered to as knockout mice (KO) and have been described previously [41]. Littermates with any of the following genotypes- Ctnnb1^loxp/loxp^;Alb-Cre^−/−^, Ctnnb1^loxp/WT^;Alb-Cre^+/−^, Ctnnb1^loxp/WT^;Alb-Cre^−/−^ are referred to as wild-type or WT. These mice are in C57BL/6 background.

Male KO (n = 9) and WT (n = 15) mice were injected intraperitoneally with DEN (Sigma-Aldrich, Inc.) at a dose of 5 µg/gram body weight at postnatal day 14 and from day 28 onwards the drinking water available *ad libitum* contained phenobarbital (PB) (0.05% w/v). Water containing fresh PB was prepared weekly for the duration of the studies. Mice were sacrificed at 8 months and liver collected for histology and protein analysis.

*Western blot Analysis:* Total tissue lysates prepared in radio immuno-precipitation assay (RIPA) buffer containing 1% IgePAL CA-630, 0.5% Sodium Deoxycholate, 0.1% SDS in 1× PBS along with protease & phosphate inhibitor (1∶100) (Thermo Scientific). Proteins were resolved on sodium dodecyl sulfate polyacrylamide gel electrophoresis in 4–15% gels and then transferred to Immobilon-P membranes (Millipore, Bedford, MA) in transfer buffer containing 10% methanol. Membranes were probed with primary antibodies (see below) in Tris-buffered saline with Tween-20 containing 5% nonfat milk or BSA. Horseradish peroxidase–conjugated secondary antibodies were used at 1∶50,000 dilution and signal assessed with Super Signal West Pico chemiluminescence substrate (Pierce, Rockford, IL) and autoradiography. The films (Molecular Technology Sales, St. Louise, MI) were scanned to obtain integrated optic densitomery (IOD) using NIH Imager software. The average IOD for a protein was compared between the KO and WT groups and assessed for statistical significance by student *t* test and p<0.05 was considered significant.

*Antibodies.* Primary antibodies used for Western blotting included: c-Met (1∶500), phospho-Met Tyr1234/1235 (1∶1000), EGFR (1∶1000), phospho-PDGFRα Tyr849 (1∶1000), phospho-IGFR (1∶1000) all from Cell Signaling Technology; β-catenin (1∶1000) from BD Biosciences; PDGFRα (1∶180), PDGFRβ (1∶500), phospho-EGFR (1∶500), phospho-PDGFRα Tyr720 (1∶200), c-myc (1∶200), PDGFA (1∶200), PDGFB (1∶200), PDGFC (1∶200), phospho-PDGFRα Tyr754 (1∶200), Glutamine Synthethase (1∶200), and GAPDH (1∶1000) all from Santa Cruz Biotechnology; phospho-PDGFRα Tyr572/574 (1∶800, Invitrogen); Cyclin D1(1∶200, Neomarkers); alpha smooth muscle actin (1∶300, Abcam); and β-actin (1∶2000, Millipore).

### Histology and immunohistochemistry

Four-micron sections of paraffin-embedded liver tissues were used for histology and immunohistochemical staining. Hematoxylin and Eosin (H&E) staining was employed to identify tumor foci. Typically four representative lobes from each mouse were included on each slide. Tumor foci were identified based on characteristic attributes such as basophilic cytoplasm and mitotic figures. For comparison, total number of microscopic nodules were counted and average numbers compared for statistical significant by student *t* test with p<0.05 considered significant.

For immunohistochemistry, antigen retrieval was achieved both by steam cooking or boiling the slides in microwave in citrate buffer for 20 minutes or 10 minutes, respectively. The sections were inactivated for endogenous peroxide, blocked and incubated with primary antibody overnight at room temperature or for one hour at room temperature, washed and incubated with appropriate biotin-conjugated secondary antibody for 30 minutes. Sections were washed, incubated with ABC reagent, washed and incubated with DAB. Sections were next counterstained with Shandon hematoxylin solution (Sigma) and cover slipped using Cytoseal XYL (Richard Allen Scientific, Kalamazoo, MI). For negative control, the primary antibody was omitted in the protocol. Slides were viewed under an Axioskop 40 (Zeiss) upright research microscope and digital images obtained. Collages were prepared using Adobe Photoshop CS4 software. For PCNA and TUNEL, numbers of positive cells were counted in four random 400× fields in five representative livers from each group and averages compared for significance between groups by student *t* test. P value of less than 0.05 was considered significant. For quantitative analysis of β-catenin- and PDGFRα-positivity in tumors, all microscopic foci in KO and WT livers were assessed. Any tumor foci showing cytoplasmic and/or nuclear β-catenin staining were labeled as being β-catenin-positive and any foci exhibiting cytoplasmic staining for PDGFRα as compared to surrounding non-tumor areas were labeled as being PDGFRα-positive. This enabled us to calculate percentage of β-catenin and/or PDGFRα-positive tumors in each group after DEN/PB treatment.

### Cell culture and treatment

Hep3B cells (ATCC) were grown to 100% confluence in 10% FBS and EMEM media. Cells were trypsinized and plated in 6 well plates containing 2 ml of 10% FBS EMEM media and serum-starved overnight. Transfection was performed using pre-validated β-catenin and control siRNA (Ambion) using lipofectamine as described previously [34]. 24 hours after transfection, cells were treated with 10 ng/ml of recombinant PDGF-CC (R&D systems) or HCl (used to reconstitute PDGF-CC) in 4% media containing tritiated thymidine. After 24 hours, the media was discarded and 1 ml of 5% trichloroacetic acid (TCA) was added to each well and plates placed in 4°C for 15 minutes. Cells were washed, dried and suspended in 1 ml of 0.33 M NaOH for 20 minutes, and 300 µl of total mixture from each well was added to 3 ml of scintillation fluid and then placed in scintillation counter. Experiment was repeated at least twice and each condition was done in triplicates. Average counts per minute (CPM) were compared between different conditions for statistical significance by student t test with value of less than 0.05 considered significant.
